# There Is No Place Like Home: A Meta‐Ethnography Exploring The Experiences of International Medical Graduates Following Return Migration to Their Home Country

**DOI:** 10.1111/tct.70188

**Published:** 2025-08-28

**Authors:** Rhiannon Newman, Mohammed A. Rashid

**Affiliations:** ^1^ School of Clinical Medicine University of Cambridge, Cambridge Biomedical Campus Cambridge UK; ^2^ London School of Hygiene and Tropical Medicine London UK

**Keywords:** doctors, international, international medical graduates, migration

## Abstract

Medical migration describes the movement of doctors across national boundaries and has historically followed a gradient from low‐ and middle‐income countries (LMIC) to high‐income countries (HIC), typically for training and income opportunities. Whilst the challenges of being an international medical graduate (IMG) are well‐documented, not all doctors stay in their host country, and comparatively little is known about the experiences of those who return to their home country. Qualitative studies examining the experiences of IMGs returning to home countries were identified through database (Ovid, ProQuest, Medline, Scopus and EBSCOhost) and manual searching and were synthesised following a meta‐ethnographic approach. A total of seven studies were included in the synthesis, published between 1975 and 2019, charting the experiences of 134 doctors returning to Africa, South America, Europe, Asia or Oceania. A total of 10 third‐order constructs were identified that were developed into overarching third‐order constructs: reflections on personal development, social and cultural connection to home countries and challenges associated with professional reintegration. Return migration in doctors has received little attention, indicating a need for further research in this area. The commonality of experiences in this review suggests that whilst family trumps finances in reasons to return, disillusionment with state healthcare systems in home countries encourages a drive to private practice and the wasted opportunity for valuable knowledge transfer. These findings can enable medical educators to better support IMGs considering and experiencing return migration, and inform policymakers seeking to optimise conditions for return migration as part of medical workforce planning.

## 1 Introduction

The flow of doctors from lower‐ and middle‐income countries (LMICs) to high‐income countries (HICs) is well‐documented; both in numbers and motivation, with financial rewards and career progression featuring strongly [1]. The consequential impact of this ‘brain drain’ of countries of origin has also been noted, sufficiently to trigger the formation of the WHO's International Platform on Health Worker Mobility, with a particular focus on countries with critical health workforce shortages [2].

The net beneficiaries of this movement have been those in the ‘Global North’—affluent nations mainly in the northern hemisphere, with similarities in socio‐economic and political characteristics [3]. Competition for medical jobs in these countries can be fierce for international medical graduates (IMGs), and medical websites, forums and even journals regularly focus on providing these doctors tips and strategies for career success [4]. This ‘all roads point west’ mentality can be seen as the natural outcome of a global cultural hegemony in medicine and medical education, where white, male, Christian, heteronormative values and experiences reign supreme [5, 6, 7]. The increasing numbers of HIC medical schools expanding to LMICs with satellite campuses and other transnational education arrangements bear testament to this trend, with teaching occurring in the English language and typically according to curricula, teaching and learning methods and assessment styles developed in the Western HICs [8, 9]. How much this contributes to the global brain drain is unquantified, but the presumed ‘portability of practices from one context to another’ has been problematised [10].

It is in this context of an increasingly globalised medical system that we consider the plight of individual doctors who have obtained their primary medical qualification from a country outside of the one where they seek to practise medicine, known as IMGs. These doctors often do not fare well in their adopted countries. Differential attainment is a phenomenon where similarly qualified candidates score differently on exams or career progression, explained by shared characteristics, such as ethnicity, gender or disability [11]. This is well‐documented in the United Kingdom and United States already, and a variety of methods have been developed to address it, including detailed studies into IMG experiences whilst in post [12, 13].

The post pandemic world is changing rapidly; the climate crisis is escalating, and politics is becoming more polarised, and the global world order is becoming increasingly ‘multipolar’ [14]. The historic pull of higher wages may cease to outweigh those of health, lifestyle and family ties, leaving the west with a potential doctor gap it may be unable to fill [12, 15].

There are now a wealth of studies investigating the push–pull factors for migrating doctors, but data on those who return are limited. A comprehensive meta‐ethnography about the experiences of IMGs in their host countries was published in 2021, synthesising over 40 qualitative studies [16]. Despite highlighting the diverse adjustments that these doctors make to succeed, studies conducted when doctors had already left were conspicuously excluded.

Not all migration is intended to be permanent, and returning home is not synonymous with failure. Indeed, one analysis has suggested that one‐quarter of all migration events result in a return migration event [17] and a changing regulatory and geopolitical landscape has renewed the focus on return migration as a concept [18]. It has also received interest in the context of mitigating healthcare ‘brain drain’ workforce policies [19]. As medical migration is a worldwide phenomenon and likely to be increasingly complex in a multipolar world, the experiences of doctors who do return warrant investigation for both positive and negative effects. In particular, it is important to understand what factors influence IMGs' decisions to return and how they experience this return. This study addresses this gap by asking: *What are the experiences of International Medical Graduates when they return to their home country following periods of medical migration?*

## 2 Methods

In examining global experiences of returning IMGs, we were interested in subjective experiences, as described by those individuals. We adopted a constructivist approach to enable us to acknowledge that experiences are true for participants at the time and in the context they were studied. To explore the experiences of doctors returning to new communities of practice [20], it felt most appropriate to seek qualitative data. Much like meta‐analysis in quantitative data synthesis, there are several approaches to synthesising qualitative data; meta‐ethnography is one such method that has been widely used in health professions education [16, 21, 22].

Meta‐ethnography is a tool to synthesise qualitative research studies and is conceptually analogous to the way in which meta‐analysis is used to synthesis quantitative research studies. It was first described by Noblit and Hare in the context of education research and seeks to ‘translate studies into one another’ [23]. They self‐described their approach as social constructivist, where knowledge is created through interaction with others and defined by the culture it exists in [23]. Since the 1980s, meta‐ethnography has increased in popularity, being widely adopted in both education and health care publications [24] and contributed significantly to the increasing acceptance of qualitative research [25]. The development of the eMERGE reporting guidelines in 2019 [26] aimed to standardise the approach to this type of study and highlight its rigorous methodology and therefore validity. Previous evaluations of meta‐ethnography have found that it is more likely to promote conceptual development than conventional narrative literature review approaches [27]. We opted to use meta‐ethnography for this reason, recognising the inevitably complexity of the topic we were studying.

**FIGURE 1:**
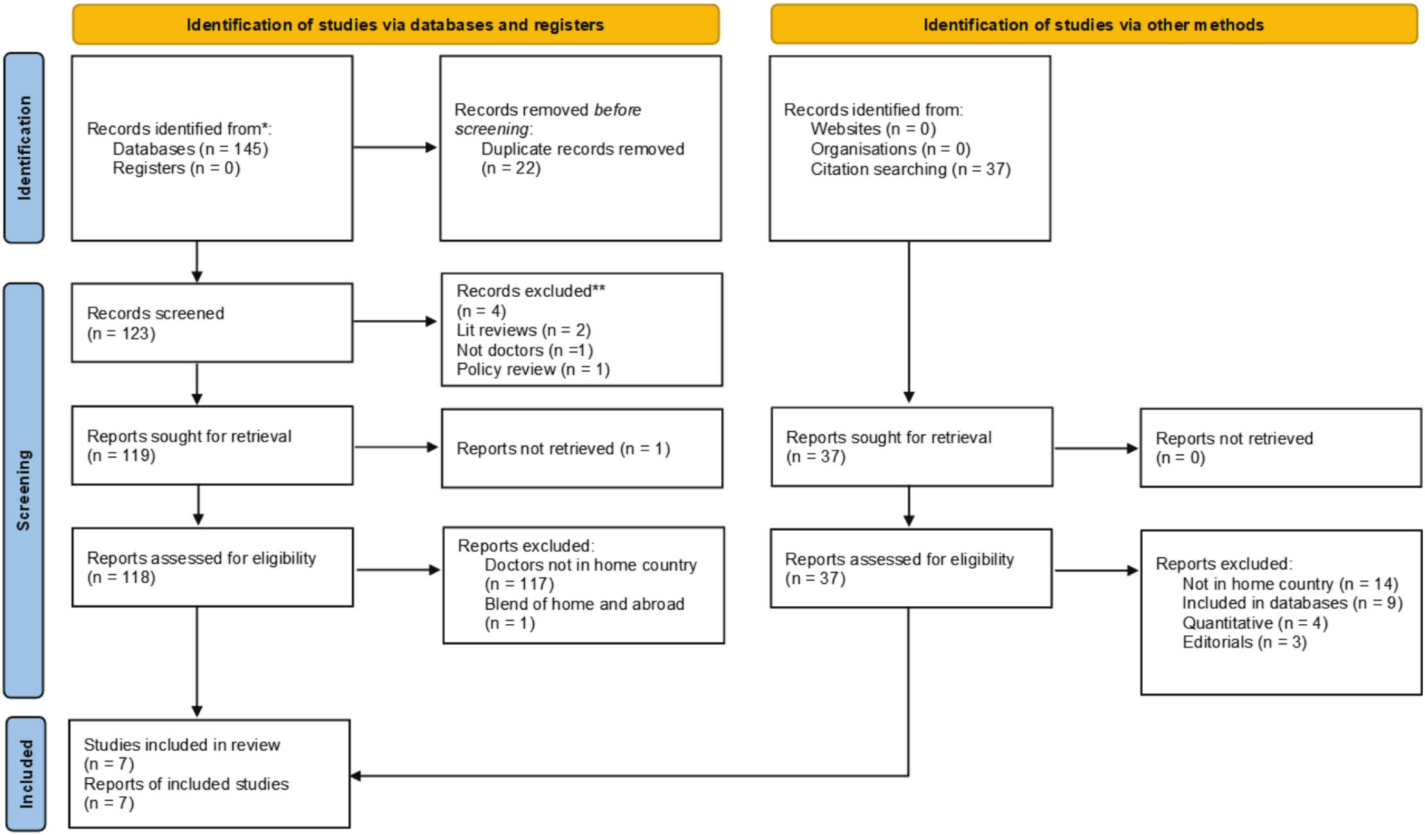
Article selection (PRISMA) flowchart.

We utilised database searches and manual searches of key reference lists and citation trackers to identify relevant studies for inclusion. Database searches were conducted in August 2023 using Ovid, ProQuest, Medline, Scopus and EBSCOhost. Search terms are shown in Table 1. No time limits were imposed, and studies were included if they used qualitative methods, included participants who were qualified doctors in their home country after a period spent working overseas, and were published in the English language. Studies that interviewed doctors in combination with other healthcare professionals were included, but those that combined interviews with participants who had not yet returned were excluded when the experiences could not be delineated. The article selection process is summarised in Figure 1 based on the Preferred Reporting Items for Systematic Reviews and Meta Analyses (PRISMA) [28]. After the removal of duplicates, all identified records were screened using titles and abstracts by both reviewers independently, who discussed discrepancies to achieve resolution. Seven articles met the inclusion criteria and were included in the meta‐ethnography.

**TABLE 1:** Terms used for database searches.

TS = (‘international medical graduates’ OR ‘foreign medical graduates’ OR ‘overseas trained doctors’ OR ‘international medical doctors’ OR ‘foreign medical doctors’) AND TS = (‘return’ OR ‘repatriation’ OR ‘retention’ OR ‘migration’ OR ‘mobility’) AND TS = (‘factors’ OR ‘barriers’ OR ‘challenges’ OR ‘motivations’ OR ‘influences’ OR ‘experiences’) AND TS = (‘qualitative’ OR ‘ethnography’ OR ‘phenomenology’ OR ‘grounded theory’ OR ‘narrative’ OR ‘thematic analysis’)

There has been debate about the value of appraisal in qualitative synthesis, with some authors suggesting articles should be judged exclusively on their conceptual merits. However, in order to optimise rigour and transparency, we opted to independently appraise all articles using the Critical Appraisal Skills Programme (CASP) qualitative research checklist [29]. Given the lack of consensus about the quality appraisal of qualitative research, we made a pragmatic decision to include all articles scoring 50% and above. As all studies met this criterion, none were excluded on the grounds of quality concerns.

The seven included studies were synthesised using a meta‐ethnographic approach. Firstly, the studies were evaluated to extract direct quotations from research participants, known as ‘first‐order constructs’ by Noblit and Hare [23]. Following this, we compiled the ‘second‐order constructs’, which were the authors' interpretations of these quotations from the original studies' results and discussion sections. We then came together to formulate their interpretations of first‐ and second‐order constructs, known as the ‘third‐order constructs’. These were developed through the ‘line of argument synthesis’, which involved identifying similarities and differences between themes to develop an overall argument that accounts for the range and diversity of the studies [23]. Studies were analysed by two researchers and discrepancies were resolved by returning to first author constructs and contextualising their reporting in manuscripts. This collaborative approach challenged our individual interpretation of constructs, decreased the possibility of biases and enabled more comprehensive understanding of these experiences [30].

We were mindful of potential impacts of our own positions as researchers as we approached this study. In that we are both medical doctors, we are professional ‘insiders’ to the phenomenon we are studying. However, whilst one of us is an IMG, neither of us has direct experience of return migration in the way we are investigating in this study, and to this degree we are'outsider'. Furthermore, we both have experience of working in international medical education and with IMGs, including many of the countries that the included studies were based in. We were cognisant of this positionality as we approached our analysis.

## 3 Results

The seven included studies are summarised in Table 2. The dates of publication range from 1975 to 2019 and covered returning doctors in South America, sub‐Saharan Africa, Europe, Pacific Islands and Asia. Four of the studies included doctors only, and the remaining three included other trained healthcare professionals amongst their participants. Each study utilised interviews, either conducted face‐to‐face, by telephone or video call. Of note, on manual searching of references, one study appeared twice—under two different titles and published in different journals with different authors listed. In this case, the earlier published article was used, having richer data in quotes and analysis.

**TABLE 2:** Summary of included studies.

First author	Year	Publisher	Country	Participants	Methods
Gaviria	1975	Journal of Academic Medicine	Peru	21 Doctors	Interviews
Williams	2008	Social Science and Medicine	Slovakia	19 Doctors	Qualitative in‐depth interviews
Connell	2008	Australian National University Press	Tonga, Samoa, Fiji	64 Clinicians, including 31 doctors	Interviews
Asampong	2013	Africa Today	Ghana	61 Clinicians, including 25 doctors	Qualitative exploratory case study
Motlhatlhedi	2018	PLoS One	Botswana	9 Clinicians, including 3 doctors	Semistructured in‐depth interviews
Heist	2019	Teaching and Learning in Medicine	Japan	19 Doctors	Semistructured in‐depth interviews
Konstantinou	2019	Keele University	Cyprus	16 Doctors	Qualitative, semistructured interview

Overall, 10 second‐order constructs were identified, as shown in Table 3, with each shown alongside an indicative first‐order construct. These 10 are organised into three categories according to the three overarching third‐order constructs identified through the line of argument synthesis.

**TABLE 3:** Summary of second and third‐order constructs.

Third‐order construct	Second‐order construct	Indicative first‐order construct
Personal development	Increased skills and knowledge	‘I had the chance to work in a very organised healthcare system and gain valuable professional experience and master my medical skills’.
	Temporary migration	‘High competition is forcing therefore doctors to gain abroad, high quality experience in order to be able to differentiate and be more competitive’.
	Entrepreneurship	‘I have been able to complete my building and opened a clinic. I see no reason why I should continue to stay abroad’.
Cultural connections	Homesick	‘Being in the UK for almost 10 years was holding me apart from my friends and relatives back in Cyprus and I was feeling home sick, missing all of my own people as well as my habits and hobbies back in Cyprus’.
	Family ties	‘My partner threatened to divorce me if I did not return to Ghana and because I cherish my marriage, I had to oblige’.
	Sense of duty	‘I've worked in Melbourne but it seems that you are doing it for the money. Here you do not get much money but you feel that you are really helping people’.
Professional reintegration	Professional jealousy	‘Those who have never been abroad scored points, saying things like “you think you are world champion.” They do not understand what I'm talking about’.
	Domestic affairs	‘I feel that patients are not given a chance to ask questions … they are not given options.… and it's very frustrating’.
	Healthcare culture	‘I never wanted to come back from Australia because it was not challenging enough but my husband wanted to return. It's not challenging. The case mixes are too few and it is not specialised here. I have to be a generalist and I do not like it. I'd like to go back there again’.
	Knowledge transfer	‘I was too young to have an impact on the system. I wasn't the chief. You have to be powerful here [the hospital] to make changes’.

The factors influencing IMG doctors' decision to return home and their experiences on return were centred around three third‐order constructs we outline. Firstly, the individual goals and achievements of the physicians played a significant role, with the majority reporting the migration was intended to be temporary, and once they had achieved their aims, return was imminent. These aims included certain levels of professional knowledge or standing or acquiring sufficient funds to invest at home. Secondly, from a wider perspective, emotional ties to family, native culture or a responsibility to one's homeland were often powerful enough to negate the potential loss of earnings or advanced healthcare practice in the host country. A third key area was reintegration into the local healthcare system. This was generally reported negatively, with returning doctors faced with professional jealousy from colleagues and inefficient systems, struggling to influence the local organisations with their imported knowledge.

Emotional ties to family, native culture or a responsibility to one's homeland were often powerful.

### 3.1 Personal Development

Personal goals were repeatedly referenced, with each study reporting that participants made definite plans on what they wished to achieve abroad and intended to return to their home countries once these were completed. Most frequently, respondents talked about using the time away to increase their professional knowledge and skill, sometimes within the host country's postgraduate training scheme:

I wanted to teach, and I was looking for a residency program where I was appreciated as a graduate of an American residency program

Even when doctors had not been involved in formal training schemes, they clearly felt that their time abroad had significantly improved their practice and capabilities, which would in turn improve both their social standing and employability on return:

It was a professional revolution for me. The best gold standard was in the Netherlands. They had different technology, and different medicines

This elevation was often translated into more prestigious jobs with resulting higher salaries, compared to colleagues who had not travelled abroad. Higher wages in host countries had often allowed doctors to save money or fund projects whilst they were away. These included both healthcare and nonhealthcare ventures, plus owning cars and property in their home countries.

To go and make a living … as being there I would afford to send money home to Botswana, to build houses I wanted … you know … there would come a stage after houses and cars and my children having gone to schools and feeling satisfied that I would then come back.

All studies reported positive experiences from time spent abroad, despite the numerous challenges inherent in practicing medicine in a country different to that of primary medical training. Sometimes these benefits were more personal than professional, such as increased self‐confidence, but there was an overwhelming feeling that individuals had achieved something good and had valuable experiences to share:

.… For me, when I came back with the skills that I had gained there.… I think that would be beneficial to Batswana ….I had worked in a big renal transplant dialysis unit.… the knowledge and the culture of appraising the evidence.

### 3.2 Cultural Connections

Motivation to emigrate has often been centred around financial drivers, which are undeniably a factor for most IMGs, moving from lower to higher income countries. Such economic arguments fall when considering reverse migration; in most studies, family connections and contact were identified as playing a highly significant role. Respondents talked about the need to care for aging parents, access affordable childcare and raise their offspring in family friendly and culturally familiar environments.

The two studies not mentioning family connections; from Slovakia and Japan, both focussed on the transfer of professional knowledge and skills, and yet both referred to culture clashes. These culture clashes occurred both in the host countries and on returning home. IMGs report experiences ranging from ‘otherness’, to being ‘aliens’ and overt discrimination or racism in both host and home countries. Nevertheless, despite frequent mention of homesickness, racism was rarely directly mentioned as a key motivator to return.

… you are not in your own country of course … it's not going to be easy … People will always remind you where you come from, they will ask you “why did you come here?” … there is that element of racism that exists … but you have to develop a thick skin. . . they will always feel like you have come to take their things…

The contrast between conditions of work in host and home countries was also commented on. Despite often advanced medical practice and technology in the host country, respondents often felt they were returning to a more manageable work‐life balance and a better quality of life. A notable exception was returning Japanese doctors, who lamented the loss of defined start and finish times they had experienced in the United States.

### 3.3 Professional Reintegration

It was mentioned that returning doctors desired to improve healthcare systems at home. The idea of knowledge transfer as a method to achieve this was reported in every paper, with doctors feeling they had ‘a wealth of experience’ to offer their home country and were eager to enact this, through medical education, management or less formal avenues. Most, however, reported incompatibility and indifference on their return.

Public healthcare systems in home countries were frequently referred to as bureaucratic, inefficient, paternalistic or even overtly corrupt. From excessive delays in starting new jobs to concerns about colleagues' work ethics or professionalism, respondents had little positive to say about government‐run systems. Sometimes their newly acquired skills were ineffectual due to a lack of technology or patient populations, and deskilling through the need to be more generalised was a common fear.

The converse was true for Japanese returnees, who sought to introduce more generalist training to an organ‐based, siloed system—yet still faced indifference or resistance to modifying the status quo. Similar frustrations around inefficiency and lack of interdisciplinary cooperation were mentioned by those returning to countries without established primary healthcare models.

It was widely reported to be difficult to implement what had been learned due to inherent attitudes around medical paternalism—from both patients and colleagues. Returning doctors worried about the lack of patient‐centred care or patient empowerment they had become accustomed to. A further complicating factor on return was the attitudes of colleagues who had remained at home. There were numerous references to professional jealousy and resentment, notably when in competition for jobs.

Overwhelmingly, returning doctors felt unappreciated by the state organisations they returned to; reports suggest a sense of incredulity that the potential gains from the knowledge transfer remained unutilised.

## 4 Discussion

This study focused on the previously underexamined topic of IMGs returning to home countries after periods of medical migration. The relatively few number of studies included reflects that this phenomenon has received little attention compared to the experiences of IMGs who are transitioning following medical migration to host countries [16]. This may reflect that it is a concern of home countries that these doctors return to, which have historically typically been from LMICs and therefore have less resources for research. A key finding of this review is that experiences are shaped by reflection on personal development, cultural connections to returning countries and challenges associated with professional reintegration. Notably, these returning doctors are not always able to create the wider impact on the healthcare system that they wish to when they return.

Experiences are shaped by reflection on personal development, cultural connections to returning countries and challenges associated with professional reintegration

Research looking into IMGs experiences in host countries reports difficulty entering training programmes, rural and isolated placements and anxiety from increasingly nationalistic politics [13, 15, 16, 31]. That these issues are not mentioned in the context of studies in this review could suggest an element of recall bias, where past concerns feel less important in the present.

The papers included in this review cover many corners of the globe and the location of the interviews is likely to have some impact on the answers given. It is worth considering that conducting these interviews in the home countries may have influenced the candidness of responses. Notably, in a study from Botswana, one participant withdrew due to concerns around confidentiality and the impact on their career [32]. In another paper, return migration was conceptualised as a failure [33]. When speaking postreturn, there is the possibility of respondents ‘saving face’ regarding reasons for coming home, such as reporting that the move was always planned.

It is surprising that no papers were identified that investigated the return experiences of Indian doctors, or more generally from the Indian subcontinent. India is the largest net exporter of doctors [34]. Data on this topic are likely to exist, perhaps on social media, forum discussions or blog posts. It would be important in future research to identify and examine this information, to establish if the themes identified here continue to be relevant to doctors from this region.

The potential implications of our findings vary according to different stakeholder groups. IMGs considering return migration may reflect on some of the potential positive and negative experiences reported in this study and to mitigate against some identified risks. Medical educators in all countries may use these findings to better support IMGs who are either contemplating or actively pursuing return migration to improve transition experiences. Finally, policymakers seeking to pull levers to influence and manage medical migration may use these findings to find mechanisms to allow returning IMGs to better integrate into the workforce and specifically to use additional skills gained overseas to improve healthcare and education systems and practices. This may include, for example, structured mentorship programmes to address the professional integration challenges highlighted in this study.

IMGs considering return migration may reflect on some of the potential positive and negative experiences

An important limitation of this research is the exclusive use of the English language. This has the potential to exclude unknown amounts of data. A multinational and multilingual team of researchers could build on the insights found through this study; this should be a priority for the advancement of knowledge in this area. It is also possible that our search strategy missed papers. Moreover, the lack of geographical diversity meant that international comparisons were not possible but should be prioritised in future research. The relative lack of prominence given to issues such as racial discrimination, ethnicity, gender, sexuality and religion in included studies was also noteworthy. These characteristics can play a key role in feelings of social acceptance in both home countries and abroad, so their influence on migration decisions is surprising and warrants further examination.

## Conflicts of Interest

The authors declare no conflicts of interest.

## Data Availability

Data sharing is not applicable to this article as no new data were created or analyzed in this study.
